# Gene cloning, expression, and characterization of two endo-xylanases from *Bacillus velezensis* and *Streptomyces rochei*, and their application in xylooligosaccharide production

**DOI:** 10.3389/fmicb.2023.1292726

**Published:** 2023-12-19

**Authors:** Jing Zhang, Yan Qin, Qingyan Wang, Sijia Liu, Jin Zhou, Baoxiang He, Xinquan Liang, Liang Xian, Junhua Wu

**Affiliations:** ^1^College of Light Industry and Food Engineering, Guangxi University, Nanning, China; ^2^National Key Laboratory of Non-food Biomass Energy Technology, Guangxi Academy of Sciences, Nanning, China

**Keywords:** xylobiose, xylooligosaccharide, endo-xylanase, sugarcane bagasse, corncob, bamboo

## Abstract

Endo-xylanase hydrolyzing xylan in cellulosic residues releasing xylobiose as the major product at neutral pH are desirable in the substitute sweeteners industry. In this study, two endo-xylanases were obtained from *Streptomyces rochei* and *Bacillus velezensis*. SrocXyn10 showed the highest identity of 77.22%, with a reported endo-xylanase. The optimum reaction temperature and pH of rSrocXyn10-Ec were pH 7.0 and 60°C, with remarkable stability at 45°C or pHs ranging from 4.5 to 11.0. rBvelXyn11-Ec was most active at pH 6.0 and 50°C, and was stable at 35°C or pH 3.5 to 10.5. Both rSrocXyn10-Ec and rBvelXyn11-Ec showed specific enzyme activities on wheat arabinoxylan (685.83 ± 13.82 and 2809.89 ± 21.26 U/mg, respectively), with no enzyme activity on non-xylan substrates. The *V*max of rSrocXyn10-Ec and rBvelXyn11-Ec were 467.86 U mg^−1^ and 3067.68 U mg^−1^, respectively. The determined *K*m values of rSrocXyn10-Ec and rBvelXyn11-Ec were 3.08 g L^−1^ and 1.45 g L^−1^, respectively. The predominant product of the hydrolysis of alkaline extracts from bagasse, corncob, and bamboo by rSrocXyn10-Ec and rBvelXyn11-Ec were xylooligosaccharides. Interestingly, the xylobiose content in hydrolysates by rSrocXyn10-Ec was approximately 80%, which is higher than most reported endo-xylanases. rSrocXyn10-Ec and rBvelXyn11-Ec could be excellent candidates to produce xylooligosaccharides at neutral/near-neutral pHs. rSrocXyn10-Ec also has potential value in the production of xylobiose as a substitute sweetener.

## 1 Introduction

Xylooligosaccharides (XOSs) are oligomers composed of β-D-xylopyranosyl (xylose) units with β-(1,4)-xylosidic linkages, with a degree of polymerization of 2–7 (Kaur et al., 2023). Prebiotic properties of XOSs, including reduced blood cholesterol and colon cancer risk; increased calcium absorption, antioxidant effects, and intestinal functions; and the medical benefit of cytotoxic activity on human leukemia cells (Amorim et al., 2019; Yegin, 2022). XOSs also benefit patients with type two diabetes mellitus by acting as substitute sweeteners without increasing blood sugar (Yan et al., 2022). Among the XOSs, xylobiose displays the minimum degree of polymerization. Xylobiose is considered the most important XOS because of its high rate of consumption by probiotics and pronounced sweetness (Li et al., 2020).

XOSs are produced from xylan in cellulosic materials, chemically and by enzyme catalysis. The typical chemical approach is autohydrolysis, which involves the acid lysis of xylan at high temperature (Surmeli and Sanli-Mohamed, 2023). Drawbacks include high energy cost for maintaining high temperature, erosion of equipment by acid, low purity of the resulting product since other polymers are also hydrolyzed, and difficulty in controlling the degree of polymerization of xylose-based products (Wu et al., 2023). The benefits of enzyme hydrolysis of xylan include specific hydrolysis of only xylan and selective control over the degree of polymerization of the produced XOSs (Madhavan et al., 2017). Furthermore, compared to the chemical approach, enzyme hydrolysis has a positive environmental impact due to the relative low temperature of hydrolysis and lack of a high concentration of acid (Girelli et al., 2020).

An endo-xylanase that is active at neutral pHs, and capable of highly specific hydrolysis of xylan with a high ratio of xylobiose in the product is desirable in the industrial scale production of XOSs. However, endo-xylanases with these characteristics are rare. Carvalho et al. (2020) used xylanase from *Aspergillus fumigatus* M51 to hydrolyze bagasse, and reported the proportion of xylobiose in the hydrolysate was 66.42%. Su et al. (2021) used a commercial xylanase from *Trichoderma reesei* to hydrolyze poplar wood and obtained 48.19% xylobiose. Xian et al. (2019) reported an endo-xylanase from *Streptomyces ipomoeae* produced 80% xylobiose from four kinds of agricultural and forestry residual xylans, but the specific enzyme activity of the enzyme was only 197.75 ± 1.42 U/mg toward beechwood xylan. Chi et al. (2013) reported an endo-xylanase from *Streptomyces thermocarboxydus* subspecies MW8 strain hydrolyzed birchwood xylan released mainly xylobiose, but the specific enzyme activity of the enzyme was only 83.94 U/mg toward birchwood xylan. Qiu et al. (2017) reported an endo-xylanase from frozen soil produced 90% xylobiose from beechwood xylan, but the specific enzyme activity of the enzyme was only 10.41 U/mg toward beechwood xylan. Moreover, most endo-xylanases are active at acidic or alkaline pHs (Chaudhary et al., 2021; de Souza et al., 2022; Zheng et al., 2022). The requirement for the addition of acidic or alkaline substances can be a drawback, as corrosion of the equipment used in the industrial production of XOSs can occur.

Global agricultural and forestry production systems generate significant amounts of cellulosic residues. Presently, cellulosic residues are treated mainly by burning as fuel or to generate plant ash that is used as fertilizer. Both treatments lead to environmental pollution and result in economic losses (Babu et al., 2022). Sugarcane bagasse (Agarwal et al., 2022), corncob (Gandam et al., 2022), and bamboo sawdust (Nkeuwa et al., 2022) are representative residues. Sugarcane is cultivated globally in tropical and subtropical regions of many countries, including Brazil, India, Thailand, China, and others (Pan et al., 2022). The global harvested yield of sugarcane was approximately 1,900 million tons in 2022 according to the Food and Agriculture Organization of the United Nations, and sugarcane bagasse is the major byproduct of the sugar industry (Alokika et al., 2021). Corn is the second largest cereal crop globally. The United States is the world’s largest corn producer, followed by China. Approximately 300,000 tons of corncobs are generated for every one million tons of corn (Potumarthi et al., 2012). Bamboo is a readily accessible, rapidly growing, and renewable resource that is globally cultivated in tropical and non-tropical areas (Fahim et al., 2022). Bamboo is a good substitute for wood in traditional furniture making, new bio-based industries, and construction fields. Sugarcane bagasse, corncob, and bamboo contain 20–25%, 33–36%, and 19–22% xylan, respectively (Xian et al., 2019; Zhou et al., 2019). Thus, they can be used to produce XOSs, thereby converting cheap residues into high-value products.

To address the demand of industrial applications, two endo-xylanases from *Streptomyces rochei* and *Bacillus velezensis* (rSrocXyn10-Ec and rBvelXyn11-Ec, respectively) were cloned and expressed in this study. The enzymes catalyzed the production of high ratios of XOSs in the hydrolysis products from bagasse, corncob, and bamboo xylan. Especially, the high ratio of xylobiose catalyzed by rSrocXyn10-Ec implicated the enzyme as an excellent candidate for the production of sweetener.

## 2 Materials and methods

### 2.1 Material, enzymes, and chemicals

All polysaccharides and XOSs were purchased from Megazyme (Ireland). Genomic DNA of *S. rochei* and *B. velezensis* were extracted using a Rapid Extraction Kit from Sangon (China). DNA amplification was used 2× Phanta Max Master Mix from Vazyme (China). PCR products were purified with DNA Purification Kit from GeneStar (China). DNA restriction enzymes (*EcoR*I, *Xho*I, and *Hind*Ш) and T4 DNA ligase were purchased from TaKaRa Bio (China). Ni^+^-NTA bead was purchased from CowinBio (China). All other chemicals were of analytical grade and commercially available.

### 2.2 Gene cloning and expression

Two strains of bacteria with xylanase activity, *S. rochei* and *B. velezensis*, were isolated from soil and moldy cartons in Nanning (Guangxi, China), and were deposited in the China Center for Type Culture Collection (Wuhan, China) under the accession numbers of CCTCC NO. M20231433 (*S. rochei*) and CCTCC NO. AB 2023223 (*B. velezensis*), respectively. Genomic DNA of *S. rochei* and *B. velezensis* were extracted and separately used as the template for the amplification of the putative xylanase gene *SrocXyn10* (GenBank No. WP_164288018.1, from *S. rochei*) or *BvelXyn11* (GenBank No. UHD41295.1, from *B. velezensis*) discovered in the National Center for Biotechnology Information genomic database. Specific oligonucleotide primers were designed using Vector NTI software based on these gene sequences (excluding the signal peptide coding region). Primers for amplification of *SrocXyn10* were SrocXyn10-F (5′-CCGGAATTCGCGGAGGCCGCCGACACG-3′) and SrocXyn10-R (5’-CCCAAGCTTGGACGCGGTGCAGCCGCC-3′). Primers for amplification of *BvelXyn11* were BvelXyn11-F (5′-CCGGAATTCGCTGGCACAGATTACTGG-3′) and BvelXyn11-R (5′-CCGCTCGAGCCACACTGTTACATTAGAAC-3′).

The pET30a(+) vector and purified PCR products were double-digested with *EcoR*I-*Hind*Ш (for *SrocXyn10*) and *EcoR*I-*Xho*I (for *BvelXyn11*). This was followed by ligation of the isolated gene and vector with T4 DNA ligase and transformation of competent *Escherichia coli* DH5α. Putative positive colonies were identified by colony PCR and DNA sequencing. Amino acid sequence identity was assessed by the Protein BLAST program. Mega 7.0 and Genedoc software were employed for multiple sequence alignments.

Recombinant plasmids were isolated by a plasmid extraction kit and transformed into competent *E. coli* Rosetta (DE3) to heterologously express the xylanase genes. The transformed bacteria were inoculated into a fresh kanamycin-containing LB medium and shaken at 16°C and 100 rpm for 12 h with 1 mM isopropyl β-d-1-thiogalactopyranoside added to induce gene expression.

### 2.3 Protein purification and identification

The recombinant enzyme obtained through ultrasonic lysis of the induced cells was purified using Ni-NTA column affinity chromatography with a nonlinear imidazole gradient of 30, 60, 100, 200, 300, 400 and 500 mM in Tris–HCl buffer (0.1 M, pH 7.5) (Vacilotto et al., 2022), and the protein was eluted by self flow of imidazole solution under the force of gravity. The purity and molecular mass of the recombinant protein were assessed using sodium dodecyl sulfate-polyacrylamide gel electrophoresis (SDS-PAGE) (Laemmli, 1970). Subsequently, the concentration of the recombinant protein was measured using the Coomassie brilliant blue R-250 method, and a standard curve was generated using bovine serum albumin (BSA) (Smith et al., 1985).

The purified xylanase (0.02 mg) was mixed with 2% (w/v) beechwood xylan in sodium phosphate buffer and incubated at 35°C. The mixture was incubated in boiling water for 5 min to terminate the enzyme reaction after 1, 6, and 24 h. The hydrolysates were mixed with pure acetonitrile in equal proportions and analyzed by high-performance liquid chromatography (HPLC) equipment UltiMate 3,000 (DIONEX, USA) using a Phenomenex amino column (00G-4378-E0, 250 × 4.6 mm, from Phenomenex, United States) and 75% acetonitrile as the mobile phase (1 mL min^−1^). The standard curve was plotted with different concentrations of xylose (X), xylobiose (X_2_), xylotriose (X_3_) and xylotetraose (X_4_).

### 2.4 Enzyme assay

Xylanase activity was measured using the previously described 3,5-dinitrosalicylic acid method (Miller, 1959). The purified enzyme (50 μL) was added to 0.5% (w/v) beechwood xylan (350 μL) in 100 mM phosphate buffer for 10 min (at pH 7.0 and 60°C for rSrocXyn10-Ec, and at pH 6.0 and 50°C for rBvelXyn11-Ec). Afterward, 800 μL of 3,5-dinitrosalicylic acid reagent was added to the mixture to stop the reaction, followed by exposure to boiling water for 5 min. After allowing the mixture to cool to room temperature, the absorbance was measured at 540 nm and the enzyme activity was calculated using a D-xylose standard curve. The amount of enzyme that released 1 μmol of reducing sugar (equivalent to xylose) per minute was defined as one unit (U) of xylanase activity.

### 2.5 Effect of pH on xylanase activity and stability

The optimal pH for xylanase activity of the purified enzyme was determined in different buffers of pH 3.0–10.0 containing 0.5% (w/v) beechwood xylan. The highest activity was defined as 100%. pH stability was tested by incubating the enzyme in buffers from pH 3.0 to 12.0 (at 4°C or 25°C for 24 h) and measuring the remaining enzyme activity. Buffers used were 0.1 M citric-Na_2_HPO_4_ (for pH 3.0–7.0), 0.1 M Tris–HCl (for pH 7.0–9.0), and 0.1 M glycine-NaOH (for pH 8.5–12.0). The activity of untreated enzyme was defined as 100%.

### 2.6 Effect of temperature on xylanase activity and stability

The optimal temperature for hydrolysis activity was determined by incubating enzyme at a range of temperature from 30°C to 75°C. To evaluate thermostability, purified rSrocXyn10-Ec and rBvelXyn11-Ec were pre-incubated in buffers (without substrate) at 30–60°C for 15, 30, 45 and 60 min. Then, the residual activity was evaluated and the activity of unprocessed enzyme was set as 100%.

### 2.7 Effect of metal ion on enzyme activity

The effect of different metal ions on the activity of purified enzyme was evaluated by adding various cations (Li^+^, Na^+^, Mg^2+^, Al^3+^, K^+^, Ca^2+^, Mn^2+^, Fe^2+^, Fe^3+^, Co^2+^, Cu^2+^, Zn^2+^, and Sr^2+^; chloride salts) in the reaction mixture with final concentrations of 1, 2.5, 5, and 10 mM. The different concentration of metal ion in the catalytic system was obtained by shifting the ratio of 100 mM metal ion solution and 100 mM phosphate buffer [containing 0.5% (w/v) beechwood xylan] as: 40 μL metal ion solution and 310 μL buffer to obtain 10 mM metal ion, 20 μL metal ion solution and 330 μL buffer to obtain 5 mM metal ion; 10 μL metal ion solution and 340 μL buffer to obtain 2.5 mM metal ion; 4 μL metal ion solution and 346 μL buffer to obtain 1 mM metal ion, and then the enzyme activity was determined by adding 50 μL enzyme solution. The enzyme activity without any additives (substitute ion solution with water) was defined as 100%.

### 2.8 Substrate specificity and kinetic parameters

The substrate specificity of rSrocXyn10-Ec and rBvelXyn11-Ec were assayed at the optimum temperature and pH for 10 min in the presence of 0.5% (w/v) beechwood xylan, wheat alabinoxylan, sodium carboxymethyl cellulose (CMC-Na), lichenan, β-glucan, and mannan. For kinetic parameters, the specific enzyme activity on 0.5–20 g L^−1^ beechwood xylan was determined and a nonlinear regression method was used to generate the Michaelis–Menten constant (*K*m) and the maximum velocity (*V*max) values with Origin 2019 software.

### 2.9 XOS production from residues

Bagasse, corncob, and bamboo sawdust obtained from local sugar factory, wet market, and a furniture factory, respectively, were heated to constant weight then crushed and screened through a 60-mesh sieve. The alkaline extract was obtained as previously described (Xian et al., 2019). The purified xylanase (0.02 mg) was combined with 2% (w/v) alkaline-extract in sodium phosphate buffer. The mixture was incubated at 35°C. The released products from alkaline extract were analyzed by HPLC as described in section 2.3.

## 3 Results

### 3.1 Gene cloning and sequence analysis

A gene fragment consisting of 1,248 bps was amplified from the genomic DNA of *S. rochei*. The comparative analysis of amino acid sequences using the SMART online tool showed that SrocXyn10 belongs to the endo-1,4-β-xylanase glycoside hydrolase (GH) family 10, because the amino acid residues 80–336 constitute a hydrolysis functional domain of GH10. A gene fragment consisting of 555 bps was amplified from the genomic DNA of *B. velezensis*. Analysis of amino acid sequences using the SMART online tool showed that the encoded protein BvelXyn11 belongs to the glycoside hydrolase (GH) family GH11. Genes of *SrocXyn10* and *BvelXyn11* have been deposited in NCBI with accession numbers of OR500515 and OR500516, respectively.

### 3.2 Protein purification and identification

**Figure 1:**
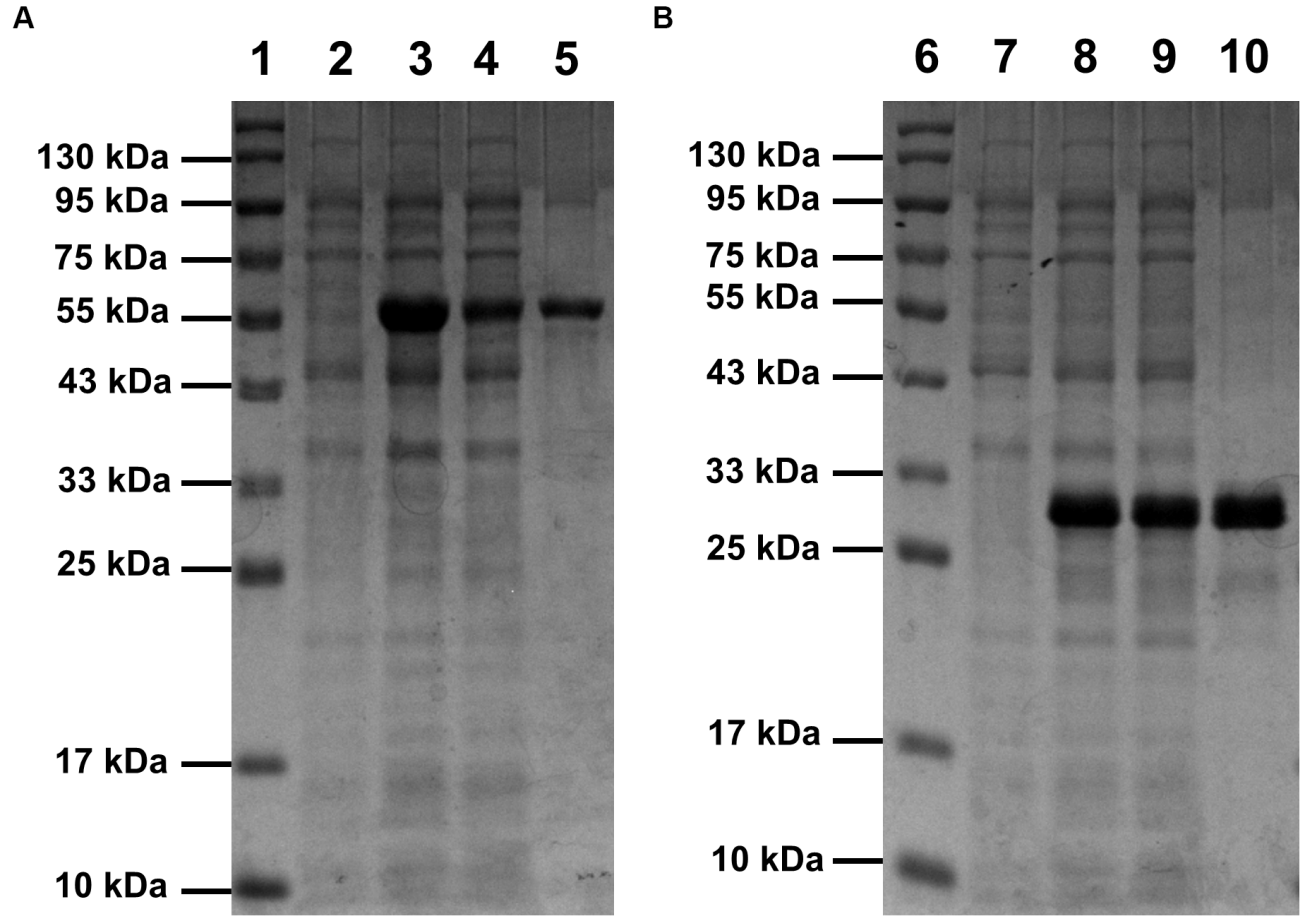
SDS-PAGE analysis of rSrocXyn10-Ec **(A)** and rBvelXyn11-Ec **(B)**. The recombinant enzymes were one-step purified using Ni^2+^-affinity chromatography. Lanes 1 and 6, protein molecular weight maker; lanes 2 and 7, cell of induced *Escherichia coli* Rosetta (DE3) harboring vector pET30a(+); lanes 3 and 8, cells of induced *E. coli* Rosetta (DE3) harboring plasmid pET30a(+)-*SrocXyn10* and pET30a(+)-*BvelXyn11*; lanes 4 and 9, soluble intracellular protein of induced *E. coli* Rosetta (DE3) harboring plasmid pET30a(+)-*SrocXyn10* and pET30a(+)-*BvelXyn11*; lanes 5 and 10, one-step purified rSrocXyn10-Ec and rBvelXyn11-Ec.

Compared with the control (lane 2 of Figure 1A and lane 7 of Figure 1B), bands of the recombinant proteins appeared in the lanes of total protein samples of induced *E. coli* Rosetta (DE3) harboring recombinant plasmid pET30a(+)-*SrocXyn10* (lane 3 of Figure 1A) or pET30a(+)-*BvelXyn11* (lane 8 of Figure 1B). After centrifugation at 12000 rpm for 2 min, the bands of the recombinant proteins also appeared in the lanes of total soluble protein (lane 4 of Figure 1A and lane 9 of Figure 1B), indicating that the recombinant proteins existed as soluble form. The recombinant enzymes rSrocXyn10-Ec and rBvelXyn11-Ec were one-step purified using Ni^+^ affinity chromatography (Table 1). They displayed apparent molecular weights of 55 kDa (lane 5 of Figure 1A) and 27 kDa (lane 10 of Figure 1B) on sodium dodecyl sulfate-polyacrylamide gels.

**Table 1:** Purification of rSrocXyn10-Ec and rBvelXyn11-Ec.

Enzymes	Purification steps	Total protein (mg)	Total activity (U)	Specific activity (U/mg)	Yield (%)	Purification fold
rSrocXyn10-Ec	Cell free supernatant	21.05	3138.98	149.11	100	1
	Ni^+^-affinity purification	4.95	1647.95	332.94	52.49	2.23
rBvelXyn11-Ec	Cell free supernatant	21.08	25498.15	1209.59	100	1
	Ni^+^-affinity purification	5.62	11803.96	2100.35	46.29	1.73

**Figure 2:**
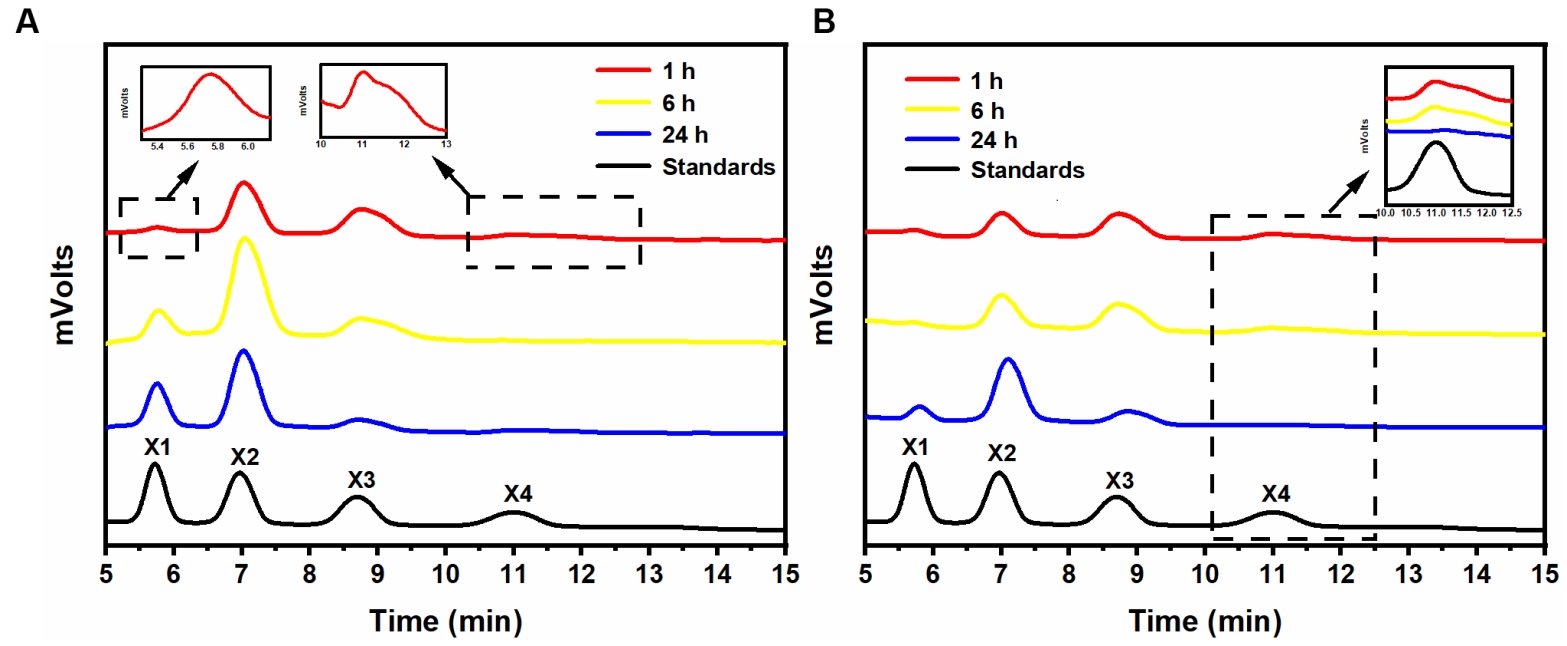
Analysis of hydrolysis products of rSrocXyn10-Ec and rBvelXyn11-Ec. HPLC analysis of hydrolysis product released by rSrocXyn10-Ec **(A)** and rBvelXyn11-Ec **(B)** for 1, 6, and 24 h.

After a 1-h hydrolysis with rSrocXyn10-Ec, the main products were xylobiose and xylotriose, with a small amount of xylotetraose (Figure 2A). Extending the hydrolysis time to 6 h resulted in undetectable xylotetraose and production of xylose. After 24 h of hydrolysis, xylose, xylobiose, and xylotriose were found in the hydrolysate. The hydrolysis behavior and products of rBvelXyn11-Ec (Figure 2B) were similar to those of rSrocXyn10-Ec. The proportion of XOSs in the hydrolysates of rSrocXyn10-Ec and rBvelXyn11-Ec were 73.94 and 89.50%, respectively.

### 3.3 Effects of pH and temperature on activity and stability

**Figure 3:**
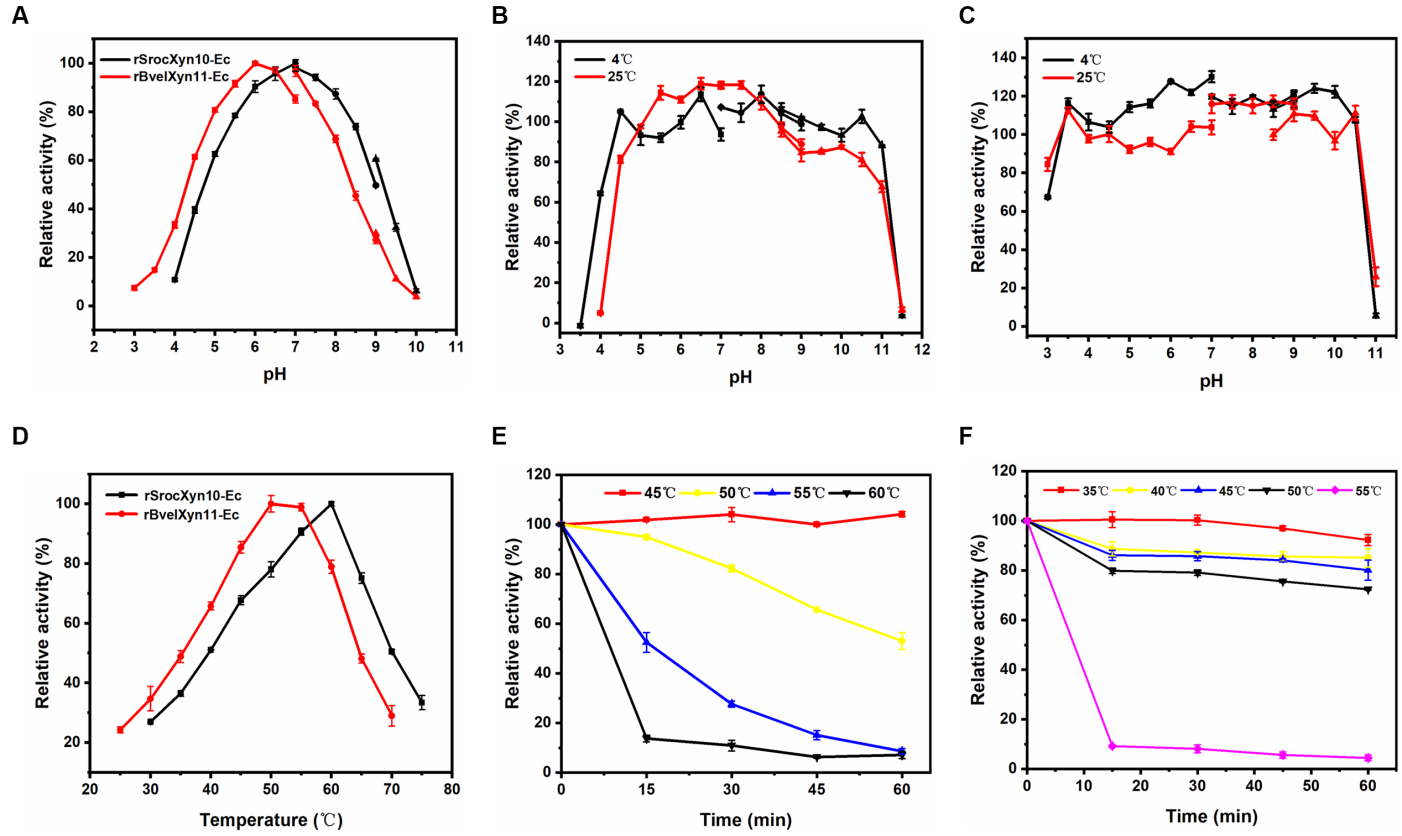
Effects of pH and temperature on activity and stability of rSrocXyn10-Ec and rBvelXyn11-Ec. **(A)** Effects of pH on enzyme activity of rSrocXyn10-Ec and rBvelXyn11-Ec. “■”: citric-Na_2_HPO_4_ (pH 3.0–7.0), “●”: Tris–HCl (pH 7.0–9.0), “▲”: glycine-NaOH (pH 9.0–10.0). (B and C) Effects of pH on enzyme stability of rSrocXyn10-Ec **(B)** and rBvelXyn11-Ec **(C)**. “■”: citric-Na_2_HPO_4_ (pH 3.0–7.0), “●”: Tris–HCl (pH 7.0–9.0), “▲”: glycine-NaOH (pH 8.5–11.5). **(D)** Effects of temperature on enzyme activity of rSrocXyn10-Ec and rBvelXyn11-Ec. **(E,F)** Effects of temperature on enzyme stability of rSrocXyn10-Ec **(E)** and rBvelXyn11-Ec **(F)**.

rSrocXyn10-Ec and rBvelXyn11-Ec showed high enzyme activity at neutral pH; the optimal pH was 7.0 and 6.0, respectively (Figure 3A). rSrocXyn10-Ec remained stable at pHs ranging from 4.5–11.0 (at 4°C) and 5.0–8.5 (at 25°C) (Figure 3B), and rBvelXyn11-Ec was stable within the pH range of 3.5–10.5 (both at 4°C and 25°C) (Figure 3C). The optimum temperature of rSrocXyn10-Ec and rBvelXyn11-Ec were 60°C and 50°C, respectively (Figure 3D). rSrocXyn10-Ec was quite stable at 45°C. The stability decreased gradually as the temperature increased. Only 10% of the original enzyme activity remained after incubation at 60°C for 15 min (Figure 3E). The thermal stability of rBvelXyn11-Ec was worse than that of rSrocXyn10-Ec. It was stable at 35°C and retained approximately 80% residual enzyme activity after 1 h incubation at 50°C. However, activity decreased significantly after 15 min incubation at 55°C, with only 9.18% residual activity (Figure 3F).

### 3.4 Effect of metallic cations

**Figure 4:**
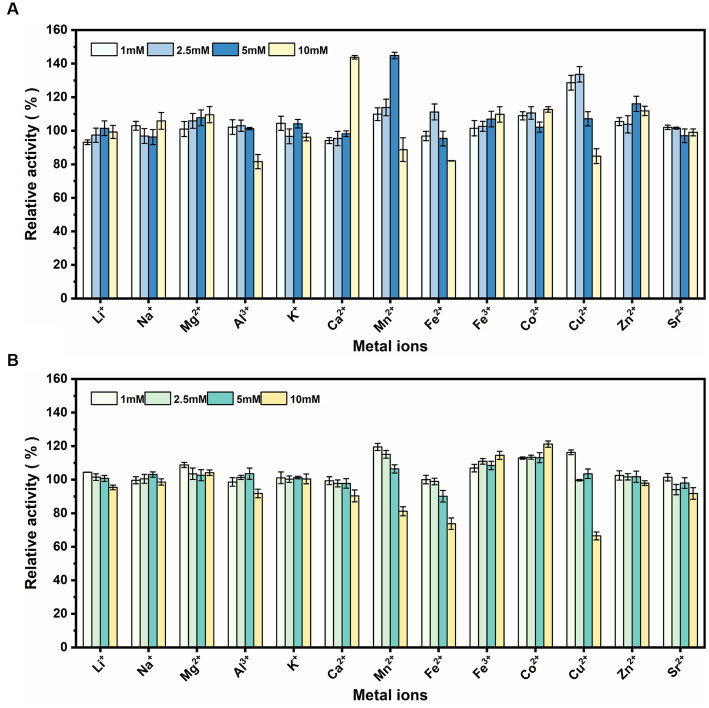
Effect of various metallic cations. Effect of various metallic cations on rSrocXyn10-Ec **(A)** and rBvelXyn11-Ec **(B)**.

rSrocXyn10-Ec and rBvelXyn11-Ec did not exhibit any obvious inhibitory influence in the presence of Li^+^, Na^+^, Mg^2+^, Al^3+^, K^+^, Zn^2+^, and Sr^2+^ (Figure 4). Ca^2+^ at 10 mM most obviously promoted rSrocXyn10-Ec; the enzyme activity was increased to 143.81% (Figure 4A). Mn^2+^ also increased the enzyme activity of rSrocXyn10-Ec at 5 mM (144.85%), but inhibited enzyme activity at 10 mM. Likewise, a low concentration of Cu^2+^ stimulating rSrocXyn10-Ec activity, increasing the activity to 128.61 and 133.61% at 1 mM and 2.5 mM, respectively. Only 84.84% enzyme activity was detected at high concentration of 10 mM Cu^2+^. For rBvelXyn11-Ec, 10 mM Fe^3+^ and Co^2+^ slightly promoted the enzyme activity to 114.47 and 121.18%, respectively (Figure 4B). With increasing concentrations of Mn^2+^, Fe^2+^, and Cu^2+^, enzyme activity decreased. Especially, with the addition of 10 mM Cu^2+^, the relative enzyme activity was only 66.46%.

### 3.5 Substrate specificity and kinetic parameters

Both rSrocXyn10-Ec and rBvelXyn11-Ec exhibited enzyme activity against xylan, while CMC-Na, lichenan, β-glucan, and mannan were not hydrolyzed (Table 2). An enzyme with this feature can promote the hydrolysis of agricultural waste to produce pure XOSs, because only xylan is degraded. Both rSrocXyn10-Ec and rBvelXyn11-Ec exhibited higher enzyme activity against arabinoxylan than against beechwood xylan, with relative enzyme activities of 185.11% and 117.46%, respectively. The *V*max of rSrocXyn10-Ec and rBvelXyn11-Ec were 467.86 U mg^−1^ and 3067.68 U mg^−1^, respectively. The determined *K*m values of rSrocXyn10-Ec and rBvelXyn11-Ec were 3.08 g L^−1^ and 1.45 g L^−1^, respectively.

**Table 2:** Substrate specificity of rSrocXyn10-Ec and rBvelXyn11-Ec.

	rSrocXyn10-Ec		rBvelXyn11-Ec	
	Specific activity (U/mg)	Relative activity (%)	Specific activity (U/mg)	Relative activity (%)
Beechwood xylan	370.50 ± 7.47	100.00 ± 1.02	2392.21 ± 17.85	100.00 ± 1.95
Wheat alabinoxylan	685.83 ± 13.82	185.11 ± 3.71	2809.89 ± 21.26	117.46 ± 4.77
Mannan	-	-	-	-
Lichenan	-	-	-	-
β-Glucan	-	-	-	-
CMC-Na	-	-	-	-

The enzyme activities of rSrocXyn10-Ec and rBvelXyn11-Ec on beechwood xylan were defined as 100%, and the relative activity of other substrates was calculated. “-” indicates that the relative enzyme activity is zero.

### 3.6 XOS production from residues hydrolysis

**Figure 5:**
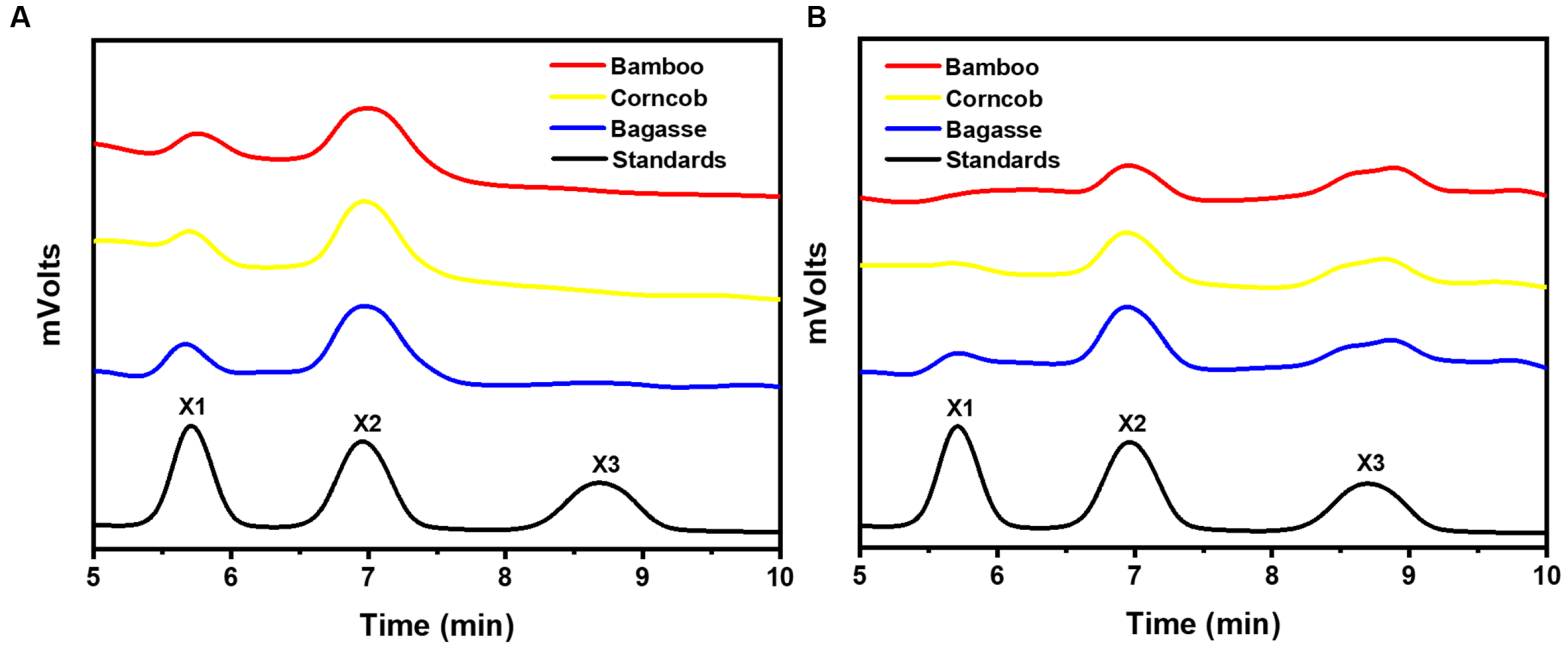
Product analysis of rSrocXyn10-Ec and rBvelXyn11-Ec from alkaline extract xylan. HPLC analysis of hydrolysis product released by rSrocXyn10-Ec **(A)** and rSrocXyn10-Ec **(B)** at 35°C for 24 h.

After 24 h of enzymatic hydrolysis at 35°C, the main hydrolysis product of rSrocXyn10-Ec and rBvelXyn11-Ec on alkali extracts of residues were both xylo-oligosaccharides (Figure 5). Notably, the major hydrolysis product of rSrocXyn10-Ec was xylobiose (Figure 5A). In more detail, they were 18.41% xylose and 81.59% xylobiose on bagasse extract, 18.21% xylose and 81.79% xylobiose on corncob extract, and 20.04% xylose and 79.96% xylobiose on bamboo extract.

The products of rBvelXyn11-Ec on residues extracts were 15.93% xylose, 49.83% xylobiose, and 34.24% xylotriose on bagasse extract; 12.87% xylose, 45.03% xylobiose, and 42.09% xylotriose on corncob extract; and no xylose, 61.24% xylobiose, and 38.76% xylotriose on bamboo extract (Figure 5B). The ratios of XOSs reached 84.07% on bagasse extract, 87.13% on corncob extract, and 100% on bamboo extract, respectively.

## 4 Discussion

**Figure 6:**
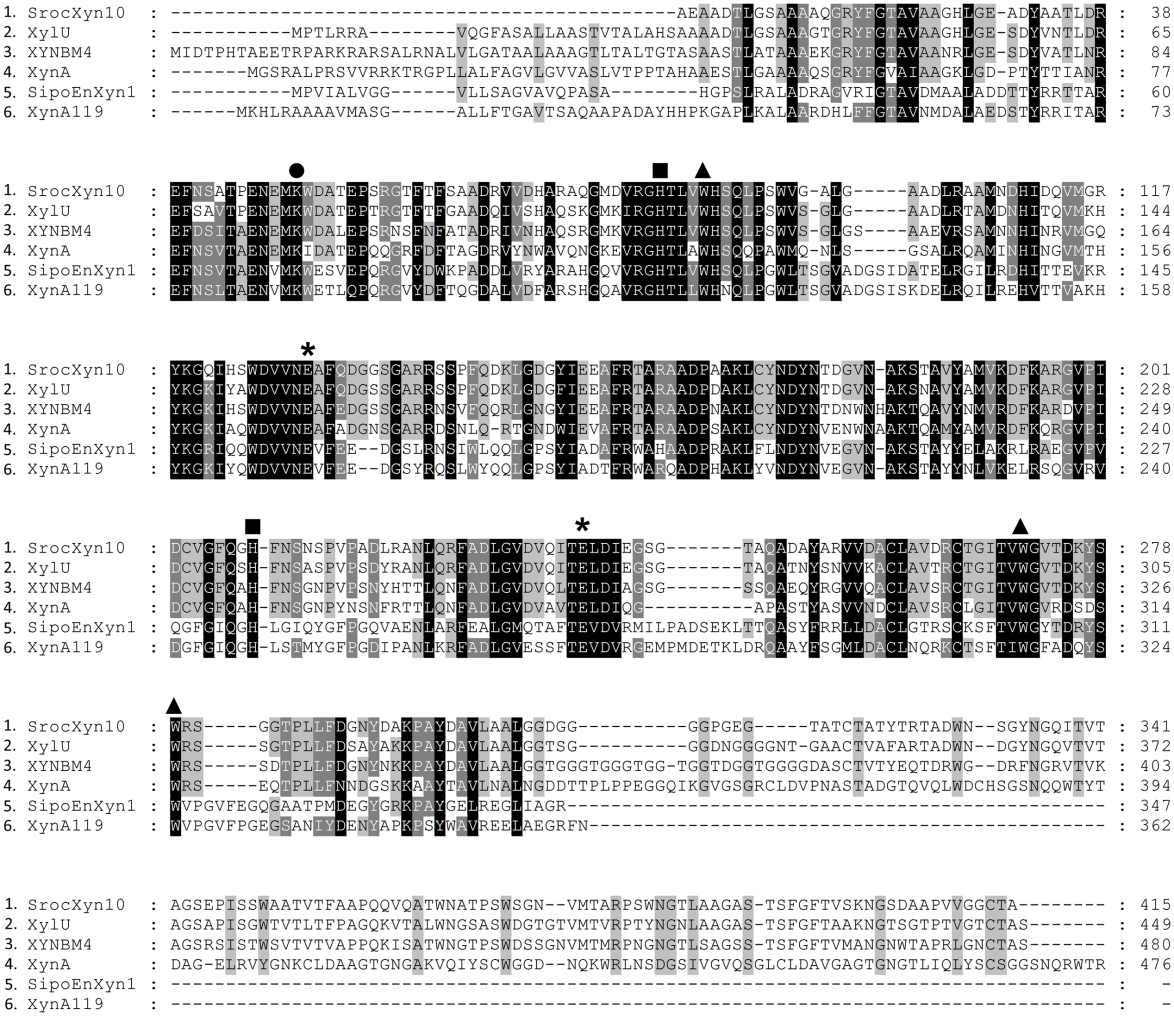
Alignment of the deduced amino acid sequence of rSrocXyn10-Ec from *Streptomyces rochei* with endo-xylanases from *Streptomyces*. XylU from *S. mexicanus* HY-14 (GenBank No: KM027334), XYNBM4 from *S. megasporus* DSM 41476 (GenBank No: HM003041), XynA from *S. thermovulgaris* TISTR1948 (GenBank No: LC088500), SipoEnXyn10A from *S. ipomoeae* 9103 (GenBank No: EKX62936), and XynA119 from *Streptomyces* sp. TN119 (GenBank No: ACR61563). Glu130 and Glu238, which were predicted to be catalytic amino acid residues, are marked by “*”. Trp87, Trp271, and Trp279, which were predicted to contribute to substrate binding, are marked by “▲”. His83 and His209, which were predicted to form hydrogen bond networks, are marked by “■”. Lys50, which was predicted to impact the *K*m value, is marked by “●”.

The encoded protein SrocXyn10 shared the highest identity of 77.22% with endo-xylanase XylU (GenBank No. KM027334) from *Streptomyces mexicanus* HY-14 (Kim et al., 2014). Sequence alignment (Figure 6) of SrocXyn10 with xylanases from the genus *Streptomyces* revealed the presence of two highly conserved glutamate residues Glu130 (acid/base catalyst) and Glu238 (catalytic nucleophile) in the conserved regions of SrocXyn10. Figure 6 illustrates the preservation of various residues, including aromatic residues, in the active site of GH10 xylanases, particularly the three tryptophan residues (Trp87, Trp271, Trp279) that actively contribute to substrate binding (Boonchuay et al., 2016). Moreover, two histidine residues (His83 and His209) play vital role in forming hydrogen bond networks, while a conserved lysine residue (Lys50) has an impact on the *K*m value (Li et al., 2017; Zheng et al., 2022). BvelXyn11 shared 100% identity with XynA (GenBank No: WP_007407578.1) from *B. subtilis* (Rhee et al., 2016). The genome sequences of species with close genetic relationships were very similar, so it is reasonable that the amino acid sequence of BvelXyn11 from *B. velezensis* was the same as XynA from *B. subtilis.* However, XynA was not tested for XOS production potential. Thus, the evaluation of XOS production potential for BvelXyn11 can be included in this study. The molecular weight of rSrocXyn10-Ec and rBvelXyn11-Ec on SDS-PAGE (55 kDa for rSrocXyn10-Ec and 27 kDa for rBvelXyn11-Ec) are consistent with the general characteristics that xylanases belonging to GH10 family show a relative high molecular weight (≥ 30 kDa), and xylanases belonging to the GH11 family share a relative low molecular weight (< 30 kDa) (Gavaseraei et al., 2021; Mendonca et al., 2023). For example, xylanase (from *B. cereus* L^−1^) (Zhang et al., 2023), Xylanase II (from *Aspergillus sydowii*) (Brandt et al., 2020) and Xyl11 (from *Trichoderma asperellum* ND-1) (Zheng et al., 2022) belonging to GH11 showed molecular weights of 23 kDa, 23.52 kDa and 22.47 kDa, respectively; and xylanase from *Penicillium menonorum* SP10 (Luong et al., 2023), AS1 (from *B. altitudinis*) (Chauhan et al., 2023) and SipoEnXyn10A (from *S. ipomoeae*) (Xian et al., 2019) belonging to GH10 showed molecular weights of 54 kDa, 43 kDa and 44 kDa, respectively. Due to the random hydrolysis of the internal β-1,4-glycosidic bond of xylan, the hydrolysis product of endo-xylanases consisted of xylose and XOSs, demonstrating that rSrocXyn10-Ec and rBvelXyn11-Ec both endo-xylanases. The high proportion of XOSs in the hydrolysates of rSrocXyn10-Ec (73.94%) and rBvelXyn11-Ec (89.50%) on commercial xylan also indicate that they might have potential value for the production of XOSs on cellulosic residues (Figure 2).

Compared with endo-xylanases with acidic or alkaline optimal pHs, the activities of rSrocXyn10-Ec and rBvelXyn11-Ec at neutral pH would be beneficial in preventing equipment corrosion by eliminating the need for acid or alkali (Tian et al., 2022). The pH stability ranges of rSrocXyn10-Ec and rBvelXyn11-Ec were broader than some xylanases, including xylanase_−_1948 (Boonchuay et al., 2016) from *Streptomyces thermovulgaris* TISTR1948 (which retains nearly 70% enzyme activity after incubation at pH 4.0–11.5) and r-XynF1 (Bhardwaj et al., 2020) from *Aspergillus oryzae* LC1 (which retains <60% activity in pH 7.0–10.0). rSrocXyn10-Ec displayed a similar optimum temperature as some xylanases from *Streptomyces* strains, such as XynSW2A and XynSW2B from *Streptomyces* sp. SWU10 (Deesukon et al., 2011), XynB-A01 from *Cohnella* sp. A01 (Gavaseraei et al., 2021), and xynAS9 from *Streptomyces* sp. S9 (Li et al., 2008).

Advantageously, rSrocXyn10-Ec and rBvelXyn11-Ec did not exhibit any obvious inhibitory influence in the presence of Li^+^, Na^+^, Mg^2+^, Al^3+^, K^+^, Zn^2+^, and Sr^2+^. Metal ions are present in plant residues. In contrast, other enzymes are inhibited by these cations (Jeong et al., 2012; Boonchuay et al., 2016; Gavaseraei et al., 2021). Metal ions bind amino acid residues on the surfaces of proteins and alter conformation and electron transport, thus affecting enzyme activity (Riordan, 1977). However, the interaction between proteins and different metal ions applied at different concentrations varies depending on the protein (Mander et al., 2014).

rSrocXyn10-Ec and rBvelXyn11-Ec share a similar property of higher activity on arabinoxylan than on beechwood xylan with the recombinant enzymes XynA (Wang et al., 2019), SipoEnXyn10A (Xian et al., 2019), and Xyn-b39 (Zhao et al., 2013). This is likely because the structure of arabinoxylans is simpler than that of beechwood (hardwood) xylans and is more easily degraded by xylanase (Wang et al., 2019). Xylose residues in sugarcane bagasse xylan are highly substituted by arabinose (Bian et al., 2012). The substrate preference of rSrocXyn10-Ec and rBvelXyn11-Ec accelerate the utilization of sugarcane waste. Notably, the *V*max of rBvelXyn11-Ec of 3067.68 U mg^−1^ is higher than most of the known endo-xylanases (Mander et al., 2014; Boonchuay et al., 2016; Xian et al., 2019; Bhardwaj et al., 2020; Brandt et al., 2020; Gavaseraei et al., 2021). The lower *K*m value of rBvelXyn11-Ec (1.45 g L^−1^) indicate the high affinity of the enzyme for the substrate and subsequent complete degradation (Roberge et al., 1999).

The proportions of xylobiose produced by rSrocXyn10-Ec (Bagasse, 81.59%; Corncob, 81.79%; Bamboo, 79.96%) were higher than that of most reported xylanases (Table 3). The high proportion of xylobiose produced by rSrocXyn10-Ec without the production of other xylooligosaccharides indicated the value of rSrocXyn10-Ec in production of XOSs and in replacing currently used sweeteners by xylobiose production.

**Table 3:** Summary of the proportion of XOS and xylobiose (X_2_) produced by different endo-xylanases.

Enzyme	Strain	Material	XOS (%)	X_2_ (%)	References
rSrocXyn10-Ec	*S. rochei*	Bagasse Corncob Bamboo	81.59 81.79 79.96	81.59 81.79 79.96	Present study
rBvelXyn11-Ec	*B. velezensis*	Bagasse Corncob Bamboo	84.06 87.13 100	49.83 45.04 61.24	Present study
Xylanase	*S. rameus* L2001	Bagasse	63.60	24.70	Li et al. (2012)
Crude xylanase	*Pichia stipitis*	Bagasse	95.30	29.80	Bian et al. (2013)
Xylanase	*G. oxydans*	Bagasse	<80.00	<35.00	Zhou et al. (2019)
Xylanase	*Aspergillus* *fumigatus* M51	Bagasse	87.36	66.42	Carvalho et al. (2020)
Xylanase	*Aspergillus foetidus* MTCC 4898	Corncob	77.08	25.00	Chapla et al. (2012)
Xylanase_−_1948	*S. thermovulgars* TISTR1948	Corncob	85.52	35.08	Boonchuay et al. (2016)
XynM	*Talaromyces amestolkiae*	Birchwood	75.22	27.08	Nieto-Domínguez et al. (2017)
XYNBM4	*S. megasporus* DSM 41476	Birchwood	NC	75.00	Shi et al. (2012)
XYL	*Aspergillus oryzae*	Poplar	87.72	29.24	Hao et al. (2020)
Commercial xylanase	*Trichoderma reesei*	Poplar sawdust	78.31	48.19	Su et al. (2021)
Commercial xylanase	*-*	Moso bamboo	67.90	31.80	Huang et al. (2019)
Xyl-gt	*Geobacillus thermoleovorans*	Wheat bran	72.96	49.65	Verma and Satyanarayana (2012)
PaXyn10A	*P. aerugineus* GY701	Hawthorn kernel	NC	<51.90	Liu et al. (2020)

NC means not calculated and “-” means no description in the reference.

The proportion of XOS produced by rBvelXyn11-Ec exceeded that of endo-xylanases from *S. rameus* L2001 (63.60% on bagasse) (Wu Q. et al., 2023), *Talaromyces amestolkiae* (75.22% on birchwood) (Nieto-Domínguez et al., 2017), *Geobacillus thermoleovorans* (72.96% on wheat bran) (Verma and Satyanarayana, 2012), and *Aspergillus foetidus* MTCC 4898 (77.08% on corncob) (Chapla et al., 2012). The ratios of xylobiose in the hydrolysates by rBvelXyn11-Ec were lower than those by rSrocXyn10-Ec. The hydrolysates of rSrocXyn10-Ec and rBvelXyn11-Ec differ in types and yields due to their distinct species and glycoside hydrolyzing family affiliations (Santibanez et al., 2021). However, high ratios of XOSs in hydrolysates by rBvelXyn11-Ec also indicated its potential for industrial scale production of XOSs.

## 5 Conclusion

In this study, endo-xylanase genes *SrocXyn10* from *S. rochei* and *BvelXyn11* from *B. velezensis* were successfully cloned and expressed. Both rSrocXyn10-Ec and rBvelXyn11-Ec were active at neutral pH, with optimal pHs of 7.0 and 6.0, respectively. Both rSrocXyn10-Ec and rBvelXyn11-Ec hydrolyzed cellulosic residue xylan with release of XOSs as the major final product. Notably, the XOS in the final product by rSrocXyn10-Ec was only xylobiose. Both rSrocXyn10-Ec and rBvelXyn11-Ec have potential value for the production of XOSs from cellulosic residues at neutral pHs. In particular, rSrocXyn10-Ec may be valuable for production of substitute sweetener xylobiose. For further research, the gene *SrocXyn10* could be heterologous expressed by food-safety host for the production of food-safety xylobiose.

## Data availability statement

The datasets presented in this study can be found in online repositories. The names of the repository/repositories and accession number(s) can be found in the article/Supplementary material.

## Author contributions

JZha: Writing – original draft. YQ: Project administration, Writing – review & editing. QW: Writing – review & editing. SL: Writing – original draft. JZho: Writing – original draft. BH: Writing – original draft. XL: Writing – review & editing. LX: Writing – review & editing. JW: Writing – review & editing.
