# Supplementary material for: Gene cloning, expression, and characterization of two endo-xylanases from Bacillus velezensis and Streptomyces rochei, and their application in xylooligosaccharide production
Source: Front Microbiol. 2023 Dec 19;14:1292726. doi: 10.3389/fmicb.2023.1292726 (PMC10762781; doi:10.3389/fmicb.2023.1292726)
Supplement: Supplementary file 1 [file Data_Sheet_1.DOCX]

**Genome Sequence**

1. **Genome sequence of *SrocXyn10* from *Streptomyces rochei* (GenBank accession number: OR500515)**

GCGGAGGCCGCCGACACGCTCGGCTCCGCTGCGGCGGCCCAGGGCCGCTACTTCGGCACCGCCGTCGCGGCGGGCCACCTCGGCGAGGCCGACTACGCCGCCACCCTGGACCGCGAGTTCAACTCGGCCACGCCCGAGAACGAGATGAAGTGGGACGCCACCGAGCCCAGCCGCGGCACCTTCACCTTCTCGGCCGCCGACCGGGTCGTCGACCACGCTCGGGCCCAGGGCATGGACGTCCGCGGCCACACCCTCGTCTGGCACTCCCAACTGCCCTCCTGGGTGGGCGCCCTGGGCGCGGCCGACCTGCGCGCCGCCATGAACGACCACATCGACCAGGTGATGGGCCGCTACAAGGGGCAGATCCACAGCTGGGACGTCGTCAACGAGGCGTTCCAGGACGGCGGCAGCGGCGCCCGGCGCAGCTCGCCCTTCCAGGACAAGCTGGGCGACGGCTACATCGAGGAGGCGTTCCGCACCGCCCGCGCCGCCGACCCCGCGGCCAAGCTCTGCTACAACGACTACAACACCGACGGCGTCAACGCGAAGAGCACGGCCGTCTACGCCATGGTCAAGGACTTCAAGGCGCGCGGCGTGCCCATCGACTGCGTCGGCTTCCAGGGCCACTTCAACAGCAACTCCCCGGTCCCCGCCGACCTGCGGGCCAATCTCCAGCGCTTCGCCGACCTCGGCGTCGACGTGCAGATCACCGAACTGGACATCGAGGGCTCCGGCACCGCCCAGGCCGACGCCTACGCGCGGGTGGTCGACGCCTGCCTCGCCGTGGACCGCTGCACCGGCATCACCGTGTGGGGCGTCACCGACAAGTACTCCTGGCGCAGCGGCGGCACCCCGCTGCTCTTCGACGGGAACTACGACGCCAAGCCCGCCTACGACGCCGTCCTGGCCGCCCTCGGCGGTGACGGCGGCGGTGGACCGGGCGAGGGCACGGCGACCTGCACGGCCACCTACACCAGGACCGCGGACTGGAACAGCGGCTACAACGGCCAGATCACCGTCACCGCGGGCAGCGAGCCGATCAGCTCCTGGGCGGCCACCGTCACCTTCGCCGCGCCGCAGCAGGTGCAGGCCACCTGGAACGCCACACCGTCCTGGAGCGGCAACGTCATGACGGCGCGGCCCAGTTGGAACGGCACGCTGGCGGCCGGCGCGTCCACCAGCTTCGGGTTCACGGTGTCCAAGAACGGCAGTGACGCCGCCCCCGTCGTCGGCGGCTGCACCGCGTCC

1. **Genome sequence of *BvelXyn11* from *Bacillus velezensis*. (GenBank accession number: OR500516)**

GCTGGCACAGATTACTGGCAAAATTGGACTGATGGGGGCGGAACAGTCAACGCAGTCAATGGATCTGGCGGGAATTACAGTGTTAATTGGTCTAATACCGGAAATTTCGTTGTTGGTAAAGGCTGGACTACAGGCTCGCCATTTAGAACAATAAACTATAATGCCGGAGTCTGGGCGCCGAATGGCAATGGATATTTGACTTTATATGGCTGGACGAGAGCACCTCTCATCGAATATTATGTAGTGGATTCATGGGGTACTTACAGACCTACCGGAACGTATAAAGGTACTGTAAAGAGTGATGGAGGTACATATGACATATATACAACGACACGTTATAACGCACCTTCCATTGATGGCGATAACACTACGTTTACGCAGTACTGGAGTGTTCGCCAGTCGAAGAGGCCGACCGGAAGCAACGCTGCAATCACTTTCAGCAATCATGTTAACGCATGGAAGAGTCATGGAATGAATCTGGGCAGTAATTGGGCTTACCAAGTCTTAGCGACAGAAGGATATAAAAGCAGCGGAAGTTCTAATGTAACAGTGTGG
